# Emulsifiers during gestation: The risks of ultra-processed food revealed in mice

**DOI:** 10.1371/journal.pbio.3002265

**Published:** 2023-08-25

**Authors:** Delphine Franssen, Anne-Simone Parent

**Affiliations:** 1 Division of Endocrinology, Diabetes and Hypertension, Brigham and Women’s Hospital and Harvard Medical School, Boston, Massachusetts, United States of America; 2 GIGA-Neurosciences, University of Liège, Liège, Belgium; 3 Pediatric Department, University Hospital, Liège, Belgium

## Abstract

Several components of our daily diet disrupt our control of energy balance. This Primer explores a recent PLOS Biology study revealing that maternal consumption of emulsifiers, commonly used as food additives, can impact the brains, metabolism and behavior of offspring.

## Teaser

Several dietary components disrupt the control of energy balance. A new study in *PLOS Biology* shows that, in mice, maternal consumption of emulsifiers induces a rewiring of the hypothalamic feeding circuits and causes neuropsychological impairment in the offspring.

The prevalence of obesity is escalating and it appears that the classic obesity risk factors of increased food intake, sedentary lifestyle, and genetics are probably insufficient to explain the rapid increase in obesity incidence. Notably, men are more commonly overweight but severe obesity is more frequent in women; these sex differences are not always sufficiently accounted for. In parallel with the rise in obesity, the consumption of ultra-processed food has been increasing over the past few decades, and recent epidemiological data have indicated that several components of such a diet may disrupt the control of energy balance and promote weight gain independently of caloric value [1,2]. In addition to sweeteners, emulsifiers, colorants, and flavorings, ultra-processed products also contain endocrine-disrupting chemicals [3]. While sweeteners in particular are known to impair metabolism and predispose to adiposity in rodent models [4], a new article in *PLOS Biology* by Mila-Guasch and colleagues [5] provides crucial information regarding the potential detrimental role of other additives, such as emulsifiers, in mice.

The Developmental Origins of Health and Disease theory originates from a historical cohort study in England that revealed a significant association between low birth weight and the occurrence of hypertension and coronary heart disease in middle age [6]. This theory, linking the risk of adult disease to early environmental conditions, initially focused on fetal exposure to nutritional restriction as a risk factor for metabolic syndrome and obesity in later life. It appears, however, that several components of maternal nutrition, not just food quantity, can affect the development of the hypothalamic circuits that control appetite and energy balance, as well as the differentiation of adipocytes or the function of the pancreas in the offspring [7]. These observations led to a paradigm shift and the development of the obesogen hypothesis, which proposes that developmental exposure to some exogenous chemicals promotes weight gain and metabolic disturbances through reprogramming of the metabolic organs. Data presented by Mila-Guasch and colleagues [5] fits into this hypothesis, as they show for the first time that maternal consumption of emulsifier alone leads to metabolic impairments in the offspring.

Food intake and energy expenditure are centrally regulated by complex interconnected neural networks within the hypothalamus. The arcuate nucleus (ARC) is the primary site for integration of hormonal (e.g., leptin, ghrelin, insulin) and environmental cues. In this nucleus, pro-opiomelanocortin (POMC) and neuropeptide Y (NPY)/agouti-related peptide (AgRP) neurons exert antagonistic effects on food intake, with POMC neurons inhibiting appetite and NPY/AgRP neurons stimulating appetite [8]. These ARC neurons project to other hypothalamic nuclei, including the paraventricular nucleus (PVN), to transmit information about peripheral energy availability. Twenty years ago, Bouret and Simerly demonstrated that these projections were established during the early postnatal period under the trophic action of leptin [8]. Not only leptin, but also ghrelin and insulin, have a role as structural organizers of the hypothalamic circuitry controlling energy balance during the first 3 weeks of life in rodents [8]. The establishment of such circuits is extremely sensitive to maternal environmental factors [9]. Postnatal overnutrition or maternal diabetes reduce the neurotrophic action of leptin during neonatal development and lead to persistent alterations of hypothalamic feeding pathways [9]. Very recent data in mice indicates that consumption of sweeteners also increases fat mass, causes glucose impairment in male offspring and decreases the density of POMC-immunoreactive fibers innervating the PVN [4]. Notably, other contaminants of modern food, such as endocrine-disrupting chemicals, have been shown to rewire hypothalamic control of appetite in a sex-specific manner [10,11].

Along this line, the study by Mila-Guasch and colleagues [5] is the first to show that maternal consumption of dietary emulsifiers disturbs the development of a hypothalamic circuit regulating energy balance in the progeny. The data suggest alterations of the maternal metabolic environment during gestation, as emulsifier-exposed mothers exhibited fasting hyperglycemia and mild glucose intolerance. At weaning, male offspring born from emulsifier-treated mothers displayed moderate glucose intolerance that persisted until adulthood. Those mice presented a delayed leptin surge during postnatal development. Transcriptional and histological analyses indicated a rewiring of the hypothalamic feeding circuits, although food intake itself was not affected. RNA sequencing analysis at weaning identified enrichment in factors regulating feeding behavior and metabolism regulation in the mediobasal hypothalamus. The authors also identified a higher staining density of α-MSH in the PVN at weaning, α-MSH being the bioactive product of POMC processing. This result was not associated with changes in the number and size of POMC neurons, but with a decrease in *POMC* and *CART* mRNA expression that could be interpreted as a compensatory mechanism. This rewiring of feeding networks was sex specific, as female offspring born from emulsifier-treated mothers did not display these alterations.

In addition to hypothalamic metabolic insults, Mila-Guasch and colleagues also brought to light the fact that maternal emulsifier exposure can lead to neuropsychological deficits, once again in a sex-specific manner. Male offspring exposed to emulsifiers showed anxiety-like traits. The combination of in utero exposure to emulsifiers with an adult Western-style diet worsened the effect in both sexes and also led to cognitive impairments in males. Neuropsychological deficits are similarly observed after early exposure to endocrine-disrupting chemicals, such as bisphenol-A (recently reviewed in [12]). The elucidation of the mechanisms of action of endocrine-disrupting chemicals and emulsifiers requires further study.

**Fig 1:**
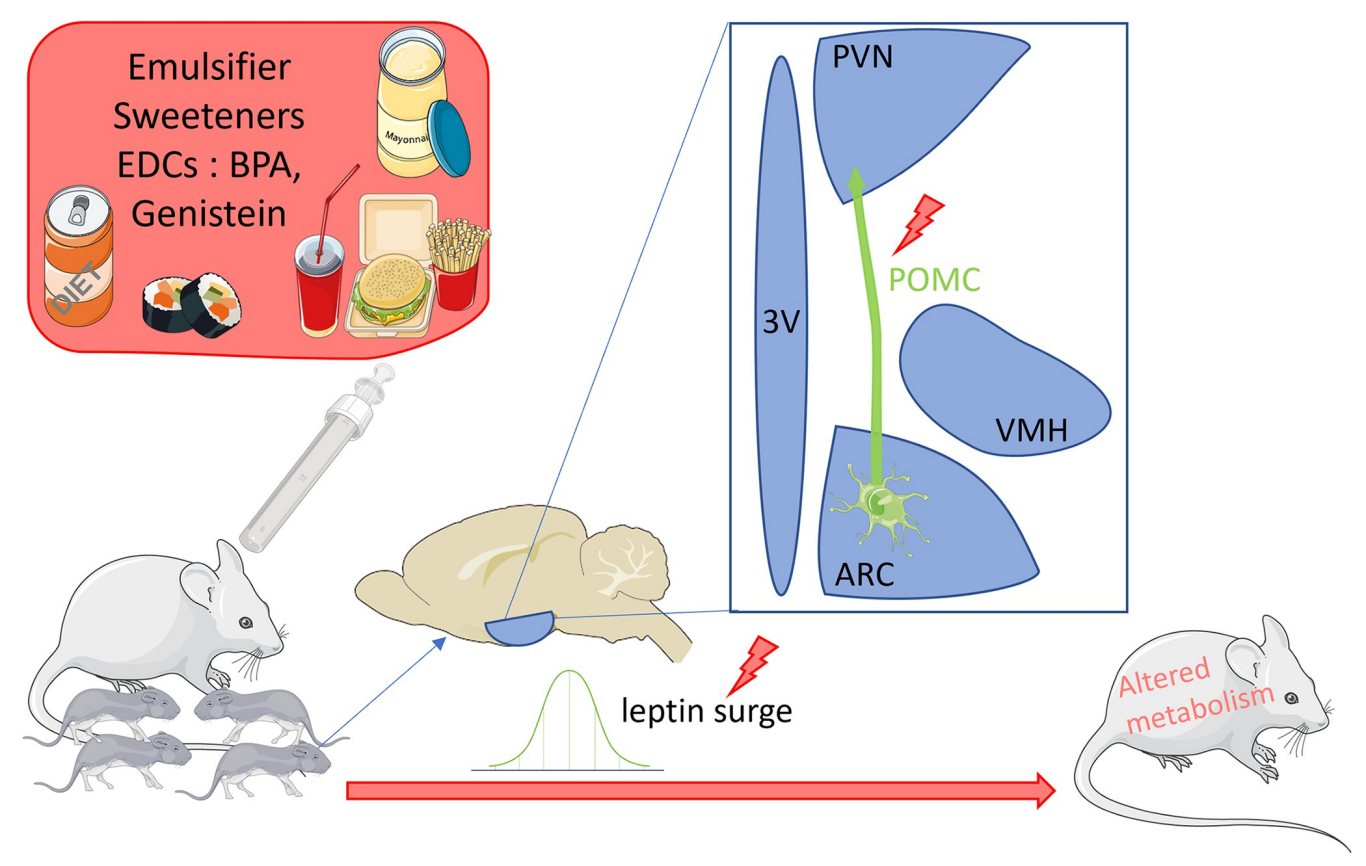
Alterations of the hypothalamic circuitry controlling energy balance after maternal exposure to compounds found in ultra-processed food. Food additives such as emulsifiers, sweeteners, and EDCs affect circuits in the hypothalamus that control energy balance in the offspring of mice exposed during pregnancy. Male offspring exposed to emulsifiers during gestation and lactation displayed moderate glucose intolerance associated with a delayed postnatal leptin surge and a higher density of α-MSH staining in the PVN [5]. Gestational exposure to sweeteners led to increased adiposity and glucose intolerance and reorganized hypothalamic melanocortin circuits in male, but not female, offspring [4]. Young male and female mice perinatally exposed to low-dose EDCs showed a delayed postnatal leptin surge during postnatal development as well as reduced density of POMC projections into the PVN. The same exposure induced glucose intolerance in adult male and hyperphagic and obesity-prone phenotype in females exposed to high-fat diet [9]. Early postnatal exposure to the phytoestrogen genistein from postnatal day 1 to 8 significantly decreased POMC immunoreactivity in females, but not in males. Those females presented an increase in body weight and altered plasma concentrations of metabolic hormones [10]. Red highlights indicate potential sites of action of food additives. Parts of the figure were drawn by using pictures from Servier Medical Art. Servier Medical Art by Servier is licensed under a Creative Commons Attribution 3.0 Unported License. 3V, third ventricle; ARC, arcuate nucleus; EDCs, endocrine-disrupting chemicals; PVN, paraventricular network; VMH, ventromedial hypothalamus.

In summary, adipose sensitivity to environmental factors is not the only mechanism that can explain an increased risk of metabolic disturbances associated with disruption of the maternal environment. Maternal nutrition also affects the development of hypothalamic circuits that control energy balance, with male offspring appearing to be more sensitive than female offspring (Fig 1). The work of Mila-Guasch and colleagues [5] shows for the first time that emulsifiers rewire the melanocortin circuits. These data reinforce the notion that compounds such as emulsifiers, sweeteners, and endocrine-disrupting chemicals that are present in ultra-processed foods could contribute to the epidemics of obesity and metabolic syndrome and suggest they may also participate in the increasing incidence of neurodevelopmental disorders. This study, even though conducted in mice, raises important public health questions, as very little data regarding food additives is present on food packaging, leading to poor information of the public. The study also suggests that limiting exposure to food additives during pregnancy may be beneficial and highlights the need for further research to examine these effects in people.
